# Maternal antioxidant provisioning mitigates pollutant-induced oxidative damage in embryos of the temperate sea urchin *Evechinus chloroticus*

**DOI:** 10.1038/s41598-017-02077-5

**Published:** 2017-05-16

**Authors:** Kathryn N. Lister, Miles D. Lamare, David J. Burritt

**Affiliations:** 1 Department of Botany, University of Otago, 464 Great King St, 9016 Dunedin, New Zealand; 2 Department of Marine Science, University of Otago, 410 Castle St, 9016 Dunedin, New Zealand

## Abstract

One mechanism of pollution resistance in marine populations is through transgenerational plasticity, whereby offspring capacity to resist pollution reflects parental exposure history. Our study aimed to establish correlations between oxidative stress biomarkers and key reproductive fitness parameters in the temperate sea urchin *Evechinus chloroticus* following exposure to dietary polycyclic aromatic hydrocarbons (PAHs). PAH-exposed adults exhibited total gonad tissue concentrations of PAHs in excess of 4 and 5 times baseline levels, for females and males respectively. Antioxidant enzymes were upregulated and oxidative lipid and protein damage to gonad tissues occurred. In addition, early stage offspring reflected maternal antioxidant status, with progeny derived from exposed females demonstrating significantly higher baselines than those derived from control females. Maternal exposure history enhanced the capacity of embryos to minimise oxidative damage to lipids and proteins following exposure to additional PAHs, but provided less of an advantage in protection against oxidative DNA damage. Abnormal embryonic development was largely independent of oxidative damage, remaining high in all embryo populations regardless of parental PAH-history. Overall, results document evidence for maternal transfer of antioxidant potential in *E*. *chloroticus*, but imply that a short-term inherited resilience against oxidative stress may not necessarily translate to a fitness or survival gain.

## Introduction

**Figure 1:**
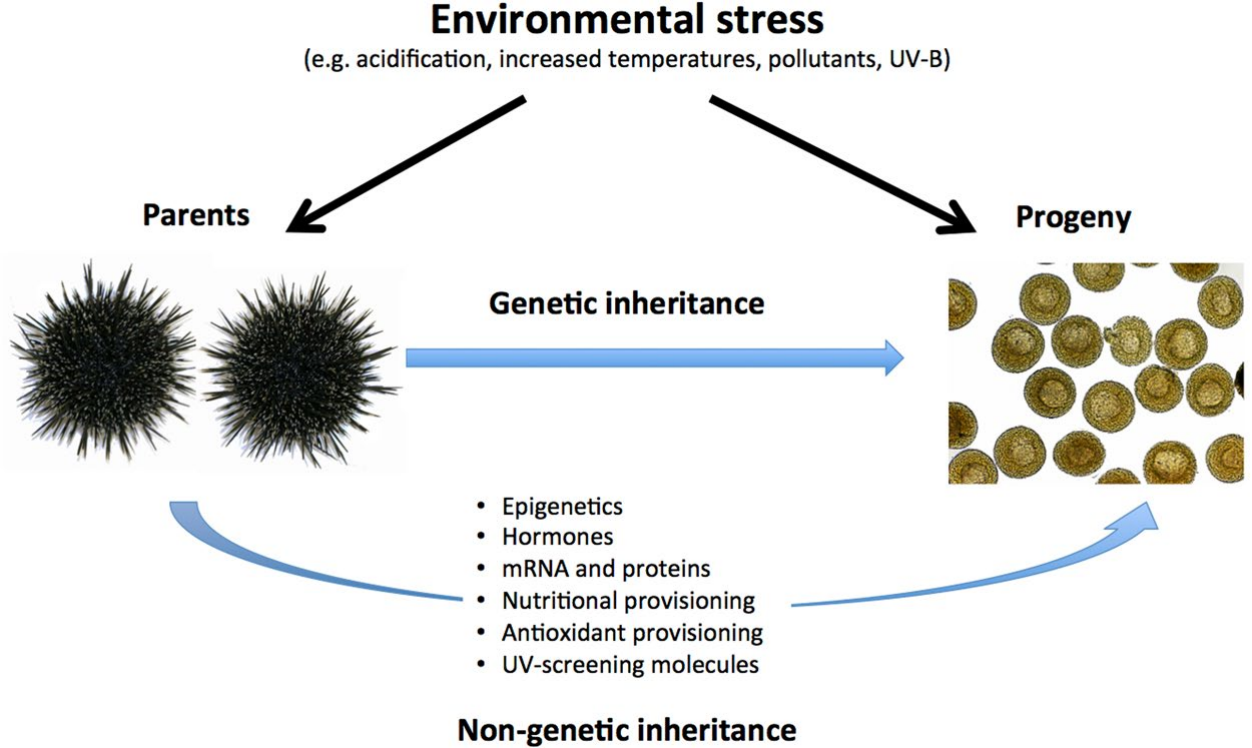
Parents can influence the ability of their progeny to respond to environmental stress through genetic and non-genetic pathways. Modified from ref. 4.

The phenotype of an animal is not only influenced by its genotype and the physical environment in which it lives, but also by interactions with other organisms^1^. With respect to the early life stages of many animals, the first and most critical interactions are with their parents. It is well documented that environmental factors can either positively or negatively influence the reproductive fitness of both maternal and paternal parents and that this can determine if their offspring live or die, especially if they are to develop in a stressful environment^2^. While the acclimation of physiological processes most commonly occurs within a generation, parental effects on offspring that are not driven specifically by offspring genotype can influence stress acclimation between generations. This phenomenon of non-genetic inheritance is often referred to as transgenerational acclimation or transgenerational plasticity and can involve the transfer of processes between generations such as hormonal changes, nutritional provisioning or epigenetics (Fig. 1)^3, 4^. Physiological responses to stress thus form one key pathway likely to contribute to the division of resources observed in reproductive trade-offs^5^, however little is actually known about the mechanisms that determine their nature and outcome, or indeed constrain the evolution of particular life history strategies^6^. The past decade has seen an increase in evidence suggesting that the capacity of individual organisms to withstand oxidative stress plays a key role in stress tolerance, and there is some evidence for transgenerational antioxidant provisioning^6–8^.

Most marine organisms are broadcast spawners, releasing their gametes into the seawater for external fertilisation to occur, and as a consequence there is no subsequent parental care^9^. For such organisms maternal provisioning is critical for the survival of any offspring and numerous studies have demonstrated the importance of provisioning eggs with sufficient energy reserves to sustain embryos/larvae until they reach a developmental stage at which foraging for external food sources is possible^10^. Few studies have investigated the importance of provisioning eggs with other specialized resources, such as antioxidants, that could help offspring cope with specific environmental stressors, such as pollutants. Antioxidant defence systems may be important drivers of life history strategies because they play a critical role in preventing damage from reactive oxygen species (ROS) to macromolecules, and are known to be involved in sexual signalling and regulating embryo provisioning^11^. Research investigating the link between reproduction and oxidative stress has so far been limited to a small number of vertebrate studies, as reviewed by ref. 12, and the impacts of ROS on life-history evolution across a greater range of taxa remains to be explored.

Echinoderms are ubiquitous within the marine environment, and many members of the phyla are long-lived, numerically dominant and act as keystone species within regions they occupy^13^. Most echinoderms have an indirect life cycle whereby they develop through short-lived embryonic and larval stages before metamorphosing into a juvenile and maturing to a long-lived adult stage^9^. The larval stage is a critical period in the life-history of species with indirect development as recruitment success is primarily determined by the survival of the embryos and larvae^9^. As a result of their sensitivity to the surrounding environment, planktonic embryos and larvae have been extensively utilised as model organisms in environmental stress physiology studies^14–19^. Oxidative challenges may be considerably higher in the early life stages of most animal species given the high metabolic activities required for development^6^. Evidence to support this suggestion has been found in several studies on birds^20^, rats^21^ and sheep^22^. Given that development potentially imposes high levels of ROS it has been hypothesized that animals should have evolved comprehensive mechanisms to counteract oxidative stress during this period^23^. Despite their widespread use as model organisms, studies employing oxidative stress biomarkers in echinoderms are few^15, 18, 19, 24–26^ and our knowledge regarding the stage(s) of development at which marine invertebrate embryos start to generate their own antioxidant systems rather than relying on those that are maternally derived is limited. Furthermore, studies that have examined impacts of pollutants on a wide range of marine invertebrates rarely include a full suite of oxidative stress markers or examine the potential costs associated with the increased energy utilised for defence and repair^25–27^.

Our study aimed to establish correlations between a range of oxidative damage and defence biomarkers and key reproductive fitness parameters in the temperate sea urchin *Evechinus chloroticus*, an important keystone species endemic to coastal New Zealand^28^. This was carried out using experimental induction of oxidative stress via dietary administration of polycyclic aromatic hydrocarbons (PAHs), a group of widespread and highly toxic contaminants found in the marine environment^29^. Although PAH metabolism is generally efficient in invertebrates, a range of direct and indirect impacts can ensue including immune and endocrine system disruption, DNA damage and the production of ROS leading to oxidative stress^29^. These consequences can, in turn, elicit either subtle or strong physiological and/or phenotypic trade-offs^30, 31^. The consequences of exogenously generated ROS in this way provide an experimental framework to test hypotheses regarding the role of oxidative stress in resource allocation, physiological adaptations and ultimately life-history trade-offs. Specifically, the following questions were asked:

1. Do short-term (21 day) dietary exposures to PAHs during the peak reproductive period cause changes in oxidative stress biomarkers in adult male and female *E*. *chloroticus* gonad tissue and released gametes?
2. Is fertilisation success rate in egg populations altered when one or both parents have been exposed to dietary PAHs?
3. Are differences in oxidative stress biomarkers observed in adults exposed to dietary PAHs also observed in their gametes and progeny?
4. Does oxidative status inherited from the parents serve embryos in response to additional waterborne PAH exposure?

## Results

### PAH tissue levels and ethoxyresorufin-O-deethylase (EROD) activities

Female and male gonad tissues from animals fed a PAH-contaminated diet contained 79.5 ng/g and 85.0 ng/g DW PAHs (phenanthrene, fluorene, pyrene and benzo[a]pyrene), respectively, compared to 19.50 and 30.67 ng/g DW for female and male controls, respectively. Eggs accumulated PAHs to levels more than 3-fold greater than baseline concentrations, with eggs from animals fed a PAH-contaminated diet containing 30.7 ng/g DW, compared to 9.0 ng/g DW for control animals. Animals also contained trace levels of naphthalene, chrysene, benzo[k]fluoranthene and indeno[1,2,3-cd]pyrene. The induction of cytochrome P450 monooxygenase 1A (CYP1A), measured in terms of 7-ethoxy-resorufin-O-deethylase (EROD) activity was also used a biomarker of PAH exposure. EROD activities in the microsomal fractions of female and male gonad tissues from animals fed a PAH-contaminated diet were 5.1 pmol/min/mg protein and 3.2 pmol/min/mg protein, respectively, while no EROD activity was detected in the female and male gonads of control animals.

### Gonad and gamete antioxidant defence

Exposure to dietary PAHs resulted in significant up-regulation of all tested antioxidant defence and detoxification enzymes except glutathione reductase (GR), as well as a greater pool of the non-enzymatic antioxidant glutathione in *E*. *chloroticus* gonad tissue of both sexes (Table 1 and Table S1). Specifically, the activities of superoxide dismutase (SOD), catalase (CAT) and glutathione peroxidase (GPx) were 2-fold higher in gonad tissue of PAH-fed females and between 1.3- and 2-fold higher in PAH-fed males compared to those fed a non-polluted diet. GR, glyoxalase-I (Glx-I), glyoxalase-II (Glx-II), glutathione-S-transferase (GST) and glutathione were upregulated to a lesser extent, ranging between 1.1-fold and 1.5-fold higher in gonad tissue of PAH-exposed individuals. Both male and female urchins also experienced a concurrent reduction (11% and 7% respectively) in levels of reduced glutathione following dietary PAH-exposure. Overall, the levels of these markers were higher in females than males, both in control and PAH-fed animals, significantly so for CAT and glutathione, as well as GPx in PAH-fed animals only (Table 1 and Table S1). Eggs derived from PAH-fed females also contained significantly higher levels of all tested antioxidant defence and detoxification markers (between 1.3- and 2.3-fold higher) compared to those from females fed a non-contaminated diet, with the exception of reduced glutathione levels which remained unchanged at approximately 90%. In contrast, sperm from both control and PAH-fed males contained negligible levels of the majority of antioxidant markers, with the exception of SOD, and to a lesser extent GR and GPx (Tables 1, S2 and S3).

**Table 1:** Mean ± SE activities of superoxide dismutase (SOD), catalase (CAT), glutathione reductase (GR), glutathione peroxidase (GPx), glyoxalase-I (Glx-I), glyoxalase-II (Glx-II), glutathione-S-transferase (GST), total glutathione (GSH + GSSG), reduced glutathione (GSH), and levels of protein carbonyls (PCs), lipid hydroperoxides (LPOX) and 8-hydroxydeoxyguanosine (8-OHdG), in the gametes and gonad tissue of male and female *Evechinus chloroticus* fed PAH-contaminated versus control *Ulva pertusa*.

	Female gonad		Male gonad	
	Control (*n* = 4)	Contaminated (*n* = 4)	Control (*n* = 4)	Contaminated (*n* = 4)
***Antioxidant defense***				
SOD (units/mg protein)	127.28 ± 10.43	249.20 ± 12.12*	163.51 ± 11.76	209.96 ± 19.05*
CAT (μmol/min/mg protein)	129.16 ± 9.76	280.60 ± 17.55*	87.11 ± 5.71	172.26 ± 27.85*
GSH + GSSG (nmol/mg protein)	86.70 ± 4.66	108.03 ± 2.82*	58.56 ± 2.45	66.92 ± 4.23*
GSH (%)	87.97 ± 2.46	80.67 ± 2.64*	89.35 ± 1.96	78.62 ± 3.00*
GR (nmol/min/mg protein)	2.61 ± 0.31	3.34 ± 0.26	2.12 ± 0.31	2.62 ± 0.41
GPx (nmol/min/mg protein)	24.02 ± 1.23	52.67 ± 3.66*	19.03 ± 1.59	32.32 ± 3.87*
GST (nmol/min/mg protein)	31.50 ± 4.34	45.79 ± 3.32*	30.99 ± 3.37	40.81 ± 8.41*
Glx-I (nmol/min/mg protein)	245.89 ± 21.13	338.32 ± 34.99*	233.24 ± 28.62	284.28 ± 42.11*
Glx-II (nmol/min/mg protein)	20.65 ± 1.82	25.33 ± 2.76*	18.26 ± 1.96	25.05 ± 1.45*
***Cellular damage***				
PC (nmol/mg protein)	1.89 ± 0.09	3.23 ± 0.15*	1.91 ± 0.14	3.26 ± 0.44*
LPOX (nmol/sample)	4.03 ± 0.13	7.70 ± 0.34*	3.11 ± 0.32	5.67 ± 0.44*
	**Eggs**		**Sperm**	
	**Control (** ***n*** ** = 3)**	**Contaminated (** ***n*** ** = 3)**	**Control (** ***n*** ** = 3)**	**Contaminated (** ***n*** ** = 3)**
***Antioxidant defense***				
SOD (units/mg protein)	144.19 ± 13.07	247.73 ± 14.25*	172.83 ± 6.22	206.49 ± 25.71
CAT (μmol/min/mg protein)	126.00 ± 1.28	289.29 ± 6.99*	T	T
GSH + GSSG (nmol/mg protein)	71.62 ± 5.70	126.10 ± 3.66*	10.88 ± 0.87	13.72 ± 3.84
GSH (%)	91.77 ± 3.85	87.99 ± 5.22	100	100
GR (nmol/min/mg protein)	1.84 ± 0.21	3.82 ± 0.06*	0.85 ± 0.11	0.72 ± 0.09
GPx (nmol/min/mg protein)	25.02 ± 1.39	53.54 ± 3.59*	21.84 ± 1.74	24.55 ± 1.78
GST (nmol/min/mg protein)	37.83 ± 1.44	49.83 ± 3.77*	T	T
Glx-I (nmol/min/mg protein)	252.88 ± 10.18	330.07 ± 7.51*	6.90 ± 2.02	7.22 ± 2.76
Glx-II (nmol/min/mg protein)	22.22 ± 1.56	33.36 ± 0.46*	T	T
***Cellular damage***				
PC (nmol/mg protein)	1.51 ± 0.15	1.53 ± 0.07	1.45 ± 0.10	1.52 ± 0.05
LPOX (nmol/sample)	2.87 ± 0.31	2.96 ± 0.22	0.95 ± 0.34	0.91 ± 0.29
DNA (8-OHG/10^6^dG)	2.23 ± 0.60	3.43 ± 0.69	4.42 ± 0.69	5.96 ± 0.60

*N* = 3. An *indicates a significant difference to the control (*P* < 0.05). T = trace levels detected.

### Gonad and gamete oxidative damage

Baseline levels of oxidative damage to proteins and lipids within *E*. *chloroticus* gonad tissue as well as in unfertilised eggs and dry sperm samples were low in individuals fed a non-contaminated diet, not exceeding 1.9 nmol/mg protein and 4.0 nmol/sample, respectively. Both male and female urchins fed PAH-contaminated *U*. *pertusa* experienced significant increases in oxidative protein and lipid damage in gonad tissue (1.7- and 1.8-fold, respectively) (Table 1 and Table S4). In contrast, eggs and sperm released from PAH-fed animals maintained low levels of oxidative lipid and protein damage, not differing from the baseline levels found in the gametes of minimally PAH-exposed field animals. Gametes also experienced no change in oxidative DNA damage following dietary PAH-exposure (Table 1 and Table S4). While similar levels of protein carbonyls were found in both sexes, male urchins experienced significantly lower levels of oxidative lipid damage (in both gonad tissue and isolated sperm) and higher levels of oxidative DNA damage, regardless of dietary PAH-exposure (Table 1 and Table S4).

### Fertilisation success

The proportion of fertilised eggs was greater than 87% for all egg populations with no significant effect of parental exposure to dietary PAHs (ANOVA: *F* = 0.33_3,8_, *P* = 0.804).

### Embryonic antioxidant defence

**Figure 2:**
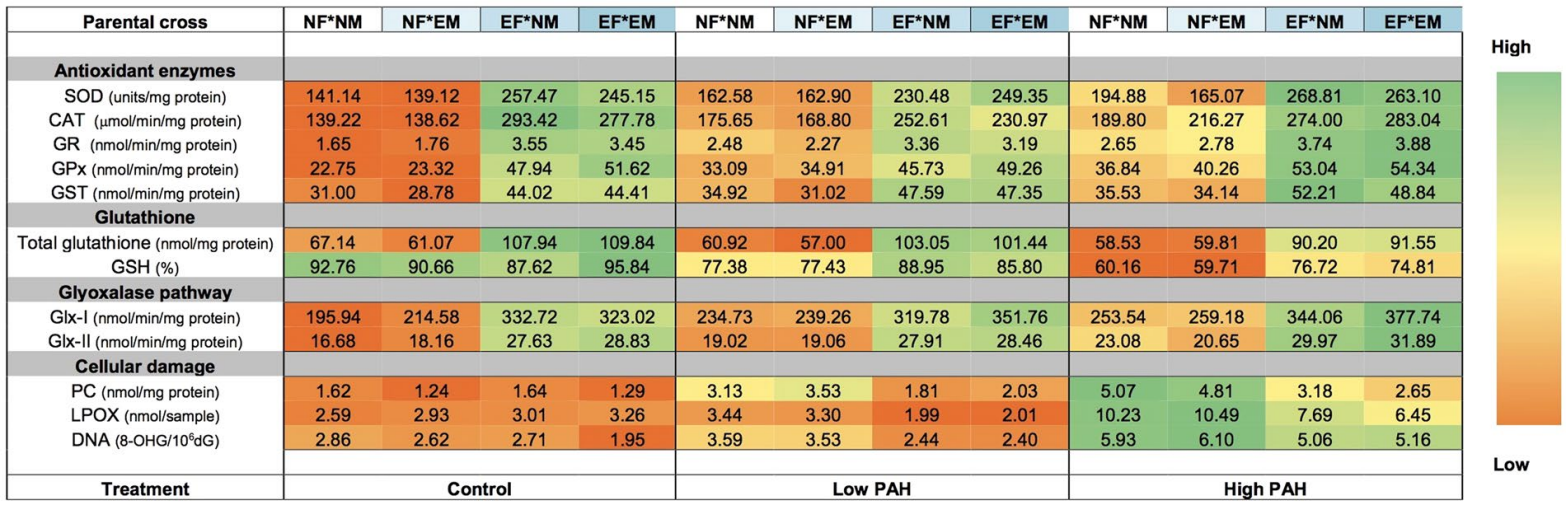
Mean ± SE activities of superoxide dismutase (SOD), catalase (CAT), glutathione reductase (GR), glutathione peroxidase (GPx), glyoxalase-I (Glx-I), glyoxalase-II (Glx-II), glutathione-S-transferase (GST), total glutathione and percentage reduced glutathione (GSH), protein carbonyl, lipid hydroperoxide and 8-hydroxydeoxyguanosine in 3-day old *Evechinus chloroticus* embryos exposed to control (0 ng/L), low (540 ng/L) and high (1080 ng/L) PAH treatments. Embryos were derived from contaminant-naïve versus contaminant-experienced parents and combinations of both (e.g. NF * NM = naïve female crossed with naïve male)﻿﻿. *N* = 3.

Antioxidant enzyme concentrations in 3-day old *E*. *chloroticus* embryos were maternally influenced with populations derived from PAH-fed mothers exhibiting higher baseline levels of all tested antioxidant defence and detoxification markers (between 1.5- and 2.2-fold higher), except reduced glutathione, than those derived from mothers fed a non-contaminated diet (Fig. 2 and Table S5). Overall, exposure to additional PAHs in the surrounding seawater at a concentration of 540 ng/L elicited negligible upregulation of antioxidant and detoxification enzymes in all embryo populations, while exposure to a higher dose of 1080 ng/L caused a small but significant increase in all markers, except GST. These increases were proportionately greater in embryo populations derived from the minimally PAH-experienced mothers collected from the field than in those derived from mothers exposed to additional dietary PAHs. In addition, embryos experienced a significant decrease in the total pool of glutathione as well as a marked alteration in glutathione redox status following exposure to additional waterborne PAHs (Fig. 2 and Table S5).

### Embryonic oxidative damage

Comparable baseline levels of oxidative protein, lipid and DNA damage were observed in *E*. *chloroticus* embryos derived from all parental crosses, with levels not exceeding 1.6 nmol/mg protein, 2.9 nmol/sample and 2.9 8-OHdG per 10^6^ dG, respectively (Fig. 2 and Table S4). Overall, a significant increase in protein carbonyls occurred following exposure to both 540 ng/L and 1080 ng/L PAHs (1.8- and 2.7-fold greater than control levels, respectively). Oxidative lipid and DNA damage was only elicited by the higher PAH dose with levels increasing 3- and 2-fold higher than control levels, respectively. In addition, embryos derived from mothers fed a non-contaminated diet experienced significantly greater damage to lipids and proteins compared to those derived from PAH-fed mothers. Oxidative DNA damage in embryos following exposure to additional PAHs in the surrounding seawater followed a similar but less pronounced trend (Fig. 2 and Table S4).

### Abnormal development

At 24 hrs post-fertilisation, the level of abnormal development was paternally influenced, with significantly greater proportions of embryo populations derived from PAH-fed fathers developing abnormally (21–28%) compared to those from dietary PAH-naive fathers (9–11%). This trend persisted following exposure to PAHs in the surrounding seawater, with no further increases in the proportion of abnormal embryos across all populations (Table 2 and Table S6). At 48 hrs post-fertilisation, the paternal effect was lost with embryos derived from PAH-fed mothers experiencing higher levels of abnormality (38–66%) than those derived from dietary PAH-naïve mothers (17–22%). This maternal influence was not present in embryos developing in PAH-contaminated water, with approximately 50% of all populations developing abnormally, regardless of parental history. At 72 hrs post-fertilisation, abnormal early larval development was significantly elevated in populations from which one or both parents were exposed to dietary PAHs. All populations developing in PAH-contaminated seawater experienced similar levels of abnormality with levels in populations derived from dietary PAH-naïve parents increasing almost 2-fold bringing them into line with populations from which one or both parents were exposed to dietary PAHs (~50%) (Table 2 and Table S6).

**Table 2:** Mean ± SE percentage abnormality in 24, 48 and 72 hr-old *Evechinus chloroticus* embryos exposed to control (0 ng/L), low (540 ng/L) and high (1080 ng/L) PAH doses.

24 hr	Parental cross			
	NF * NM	NF * EM	EF * NM	EF * EM
Control	11.20 ± 4.20^a^	20.53 ± 1.79^b^	9.87 ± 1.13^a^	27.79 ± 0.54^b^
Low	12.13 ± 3.79^a^	20.97 ± 6.28^b^	14.53 ± 1.85^a^	26.39 ± 5.27^b^
High	14.30 ± 2.55^a^	25.12 ± 4.54^b^	14.84 ± 0.51^a^	28.36 ± 5.98^b^
**48 hr**				
Control	16.94 ± 1.53^a^	21.73 ± 2.00^ab^	37.82 ± 3.04^abc^	65.63 ± 2.57^d^
Low	46.67 ± 7.03^cd^	43.55 ± 1.23^bcd^	50.30 ± 0.86^cd^	59.36 ± 3.87^cd^
High	55.33 ± 4.43^cd^	54.97 ± 3.53^cd^	54.16 ± 6.36^cd^	56.35 ± 8.58^cd^
**72 hr**				
Control	26.42 ± 2.18^a^	52.52 ± 3.52^bc^	59.27 ± 1.86^c^	48.30 ± 1.79^bc^
Low	34.18 ± 3.95^ab^	45.04 ± 3.64^abc^	58.37 ± 5.23^c^	59.18 ± 6.54^c^
High	48.16 ± 2.98^bc^	54.31 ± 0.65^bc^	42.90 ± 5.61^abc^	57.41 ± 4.90^c^

Embryos were derived from contaminant-naïve versus contaminant-experienced parents, and combinations of both (e.g. NF * NM = naïve female crossed with naïve male). *N* = grand mean of 3 counts (40–60 embryos per count). Different letters indicate significant treatment effects (*P* < 0.05) using multiple comparisons testing (Tukey’s HSD).

## Discussion

Short-term acute dietary PAH exposure induced oxidative stress in both male and female *E*. *chloroticus* adults, evidenced by an increase in oxidative lipid and protein damage to gonad tissues and an upregulation of antioxidant defences. Interestingly, EROD activity was not detectable in the gonads of control animals and only relatively low EROD activities were found in the gonads of PAH fed animals, suggesting that the reproductive tissues have only a limited capacity to detoxify PAHs via monooxygenases (MOs). For invertebrates, the highest levels of MOs have been found in tissues that are associated with the digestion of food^32^. Hence, the relatively low levels of MO activity found in this study are not unexpected and suggest that a large increase in PAH detoxification capacity is not a primary provisioning mechanism to mitigate the impacts of pollutants in early-stage *E*. *chloroticus* embryos.

In contrast, early-stage offspring show relatively large increases in maternal antioxidant provisioning, with populations derived from PAH-exposed mothers demonstrating significantly higher baseline levels of antioxidants than those derived from control mothers. In the present study, three biomarkers of oxidative damage were measured, and seven enzymes as well as glutathione were utilised as biomarkers of antioxidant defence. While it is possible that other molecules with antioxidant activity, such as ovothiol and algal pigments could play a role in antioxidant provisioning^33^, it is likely that these would follow the same trends seen for the other eight biomarkers of antioxidant defence measured. Maternal exposure history enhanced the capacity of embryos to minimise oxidative damage to lipids and proteins following exposure to additional PAHs, but provided less of an advantage in protection against oxidative DNA damage. Conversely, paternal exposure history had little influence on whether embryos were more or less susceptible to oxidative damage when exposed to waterborne PAHs, at least in the early pre-feeding stages examined. Interestingly, abnormal or delayed embryonic development in response to PAHs was largely independent of oxidative damage, remaining high in all embryo populations regardless of parental PAH history. Overall, results provide evidence for maternal transfer of antioxidants in *E*. *chloroticus*, but imply that an inherited resilience against oxidative stress may not necessarily translate to a fitness or survival gain when faced with additional contaminant stress, at least during the early embryonic development examined here.

Although a small PAH burden was found in our control population, additional acute dietary PAH exposure resulted in considerably greater concentrations in treated animals and clear biochemical effects both in adults and offspring. These baseline findings in the animals’ gonad tissue likely resulted from nearby road run-off or boating activities close to the time of collection and demonstrate the ecological relevance of PAH contamination in the marine environment. Monitoring following a significant oil spill off New Zealand’s northwest coast in 2011 documented PAH concentrations in nearby *E*. *chloroticus* gonad and gut tissue that were similar to those found in our PAH-treated animals, demonstrating that the levels we achieved experimentally were environmentally relevant^34^. An important finding in our study was that eggs carried less than half the burden of parent PAH molecules than female gonad tissue, indicating a possible exclusion mechanism. These data are interesting since *E*. *chloroticus* eggs have high lipid contents and most PAHs are lipophilic^35^. Nevertheless, even low PAH levels are likely to be problematic in eggs as they could be released slowly while the reserves are mobilised during early embryonic development. While it was beyond the scope of this study to analyse, sperm also contain high lipid levels and might be expected to be susceptible to lipophilic contaminants. PAH toxicology with respect to invertebrate germ cells has received limited attention in the literature^26, 35, 36^ and warrants further investigation.

As a result of dietary PAH exposure and uptake, greater oxidative lipid and protein damage was observed in both male and female gonad tissue despite a clear upregulation of antioxidant defenses. Critically however, eggs and sperm were largely protected, demonstrating that damage measured in the gonad was predominantly occurring in somatic tissues. In comparison, elevated, but not statistically significant, increases in DNA damage to both gametes were observed following parental dietary PAH exposure. DNA damage may be a more sensitive biomarker for pollutant stress in these organisms, as has recently been shown in avian oxidative stress research^37^. Pollution may impair reproductive success by reducing the quality of either gamete, with sperm often proving more susceptible to chemical stress than eggs^38^, although this is not always the case^35^. Sex-specific differences observed both prior to and following dietary PAH exposure in the present study included greater lipid damage to eggs but greater DNA damage to sperm, upholding only in part the greater sensitivity of sperm. These results are consistent with those found in a study of the sea urchin *Psammechinus miliaris* in which a range of biochemical parameters differed between male and female animals, but were minimally affected by exposure to the PAH phenanthrene^35^. Furthermore, sperm in the present study had low to negligible levels of antioxidants whereas eggs had considerable concentrations, and these increased markedly following dietary PAH exposure. While sperm have limited antioxidant potential due to the small cytoplasmic volume and thus contribute few resources to a developing embryo, they do contribute genetic material and high rates of DNA damage can have important effects on fertilisation success and normal embryo development^39^. Given that both male and female gametes are released prior to fertilisation in broadcast spawning organisms, and females are unable to bias investment in their eggs in response to sperm quality, paternal effects may be as likely to develop as maternal effects in these systems^40^.

No significant effect of parental PAH history on fertilisation success was observed, providing additional evidence that the populations of gametes as a whole were not negatively affected by adult PAH exposure. In contrast, a dose-response study of the sea urchin *Anthocidaris crassipina* found that sperm were damaged and more likely to reduce fertilisation success than eggs following adult exposure to cadmium^41^. The impacts of contaminants on sperm quality and subsequent fertilising capacity in marine invertebrates have been well documented^41, 42^ and it seems likely that we would have seen a paternal effect had fertilisation occurred in PAH-contaminated water, rather than under optimal lab conditions. It is also important to note that fertilisation rates can be affected by sperm densities, whereby if densities are high enough fertilisation will occur regardless of a high percentage of impairment^43^. Both the present study and that of *A*. *crassipina* ^41^ used only one concentration of sperm and direct comparison between studies must be treated with caution. When gametes are pooled from multiple parents, high rates of fertilisation can occur as a result of over-representation by the most stress tolerant male(s)^44, 45^. Nevertheless, the pooled gamete approach reflects natural conditions for broadcast spawners and provides insights into the likely population response to environmental stressors. Furthermore, successful recruitment and establishment of populations requires that all ontogenetic stages are completed successfully and successful fertilisation does not necessarily guarantee normal development or offspring longevity^44^.

There is limited literature available that examines oxidative damage and ontogenetic antioxidant defense parameters in early, pre-feeding developmental stages of marine invertebrates^15, 18, 19, 24–26^. A core hypothesis posed by our study was that embryos derived from adults potentially pre-adapted to PAH stress would either have a reduced or enhanced capacity to respond to additional PAH exposure compared with those derived from PAH-naïve parents. Although antioxidant protection was not sufficient to prevent damage from occurring across all embryo populations in response to waterborne PAHs, embryos derived from dietary PAH-naive mothers experienced proportionally greater rates of upregulation. Indeed, antioxidant activities in embryos derived from PAH-fed mothers remained more or less constant regardless of PAHs in the surrounding seawater, demonstrating that the capacity for quenching excess ROS or detoxifying toxic by-products was likely already operating at the upper limit in these embryos. The fact that oxidative damage, particularly to lipids and proteins, was considerably greater in the embryo populations generated from dietary PAH-naïve compared to PAH-experienced mothers may indicate that the higher baseline antioxidant levels in the latter populations and the reduced requirement for additional synthesis did in fact infer greater protection through energy savings. Certainly, the synthesis of protective enzymatic antioxidants is an energetically expensive process for marine invertebrates^46^.

The cost of protection and/or repair of oxidative damage can represent important resource trade-offs for broadcast spawning marine invertebrates, with any relocation of resources away from growth and development potentially influencing larval fitness. Abnormal development in *E*. *chloroticus* embryos in response to PAHs was largely independent of oxidative damage in the present study, remaining high in all embryo populations regardless of parental history. Furthermore, baseline levels of abnormality in embryo populations developing without additional exposure to PAHs changed with age making conclusions regarding the influence of parental PAH history difficult. Multiple pathways influence normal embryonic and larval development, and different genetic and physiological triggers may be responsible for our findings at each developmental stage. For example, abnormal development can be a consequence of delays in cell division, apoptosis or the breakdown of a range of cellular repair mechanisms^46^. PAHs have been shown previously to delay and disrupt normal development in echinoderm larvae^17, 26, 47, 48^, for example by causing exogastrulation through a beta-catenin dependant pathway, although the underlying mechanisms were not directly ascertained in the current study.

The levels of 8-OHdG measured in the present study are similar to those previously documented in the temperate sea urchin, *Strongylocentrotus droebachiensis*, in which baseline levels of around 1 ng/ml and rising to above 3 ng/ml following exposure to UVR, were seen^24^. However, these levels are low in comparison to other marine invertebrates in response to environmental stressors^49–51^. Although there is limited information regarding oxidative DNA damage in echinoderms, even low levels can be detrimental to development if DNA repair is not successful. If repair mechanisms fail, the affected cells in the developing embryo undergo apoptosis^46^, which may have contributed to the abnormalities observed in our study. Damaged DNA can also have longer lasting consequences than lipid and protein damage, particularly if the damage to lipids and proteins measured in the current study originated in the storage forms of these molecules. In addition, the functional integrity of DNA can also be compromised directly from exposure to certain PAHs, such as benzo[a]pyrene, which can bind to DNA and interfere with normal development^52^.

The parent-to-offspring transfer of a range of contaminant-mediated trait changes is not rare in nature^53^, and inter-generational trade-offs may be particularly important in understanding how environmental variables contribute to life history traits^54^. Maternal provisioning can have far reaching impacts on the successful development and survival of offspring in a wide range of marine embryos and larvae^55–57^, however oxidative stress is not often examined as a potential mechanism. Developmental plasticity may be due to differences in maternal loading of protective factors during oogenesis^55^ and our findings provide evidence that antioxidants are potentially strong candidates for this. In addition, maternal experience can have complex effects on offspring phenotypes, increasing performance in one life-history stage but reducing it in another. Developmental abnormalities, which reduce larval survival either directly or indirectly through slowed development, can result in considerable impacts on adult populations^58^ and can therefore have negative consequences at the population and ecosystem levels. Our study highlights that for ecologically and economically important species, such as *E*. *chloroticus*, future research must consider the long-term multigenerational effects of environmental stressors and identify sensitivities of all ontogenetic stages.

## Materials and Methods

### Sampling sites and animal collections

*Evechinus chloroticus* were collected from the Southeast coast of New Zealand (45°28′03.56′S, 170°49′48.32′E). Thirty individuals were transported back to the laboratory and placed randomly within 10 l plastic tanks (one individual per tank), each independently supplied with flowing filtered (22 µm) seawater (FSW). Tanks were arranged in a randomized block design receiving ambient light from nearby windows, and the seawater temperature was approximately 16 °C for the duration (21 days) of the experiment. Animals were acclimated to the laboratory conditions for three weeks prior to the start of the experiment and were not fed during this time to allow their gut to depurate. Urchins were measured for horizontal test diameters (HTD) (±10 mm) using vernier digital callipers to ensure all experimental individuals used were within the same age cohort (80–100 mm).

### Experimental design

Vegetative thalli of *Ulva pertusa* were collected from the Otago Peninsula, New Zealand (45°50′59.58′S, 170°42′20.15′E) by hand at low tide. The algae were then cleaned and incubated in a Contherm 620 growth cabinet set to 12 °C and provided with 375 µmol m^−2^ s^−1^ of photosynthetically active radiation (PAR) by lamps (Phillips, Aquarella) over a 12 h light/dark cycle. Algae were loaded with PAHs by incubating thalli for three days in FSW, replaced daily, containing a mixture of phenanthrene, fluoranthene, pyrene and benzo[a]pyrene (dissolved in acetone), at a ratio of 1:2:2:1, maintained at a concentration of 1080 ng/L. A second batch of *U*. *pertusa* was incubated in FSW containing the same volume of acetone (3 ml) to be fed to control animals. Urchins from 15 randomly selected tanks were fed contaminated *U*. *pertusa* and those from the remaining 15 tanks received uncontaminated *U*. *pertusa*. Animals were checked twice daily and individually fed once a week, with the same amount of food, for 3 weeks. PAH content of *U*. *pertusa* was determined for each batch and, while PAH concentrations varied between batches, animals fed PAH contaminated *U*. *pertusa* received on average a total of 1692 ng PAHs over the 3-week feeding period.

### Spawning and embryo culture

One week following the last feed, all urchins were induced to spawn in the laboratory by an inter-coelomic injection of 1–2 ml of 0.5 M KCl solution. Females were inverted over appropriate sized beakers containing FSW in order to collect eggs. After the gamete flow had stopped animals were removed and the eggs cleaned by serial partial water changes. Sperm from four ripe males from each treatment was collected dry, combined and kept in tubes on ice until needed. After gametes had been collected, each individual urchin was dissected and the gonads were removed, homogenized, snap frozen in liquid nitrogen and stored at −80 °C for later use in biochemical analyses. Three replicate concentrated subsamples of pooled eggs and sperm (~50 mg per sample) were collected in 1.5 ml Eppendorf tubes, snap frozen in liquid nitrogen and immediately stored in −80 °C for later biochemical analyses.

### Fertilisation success

The eggs from four females per treatment were combined in order to gain sufficient material for the embryo stress experiment and each egg solution divided evenly between two 1 l glass beakers. To achieve a sperm-to-egg ratio of 500:1, sperm concentrations were quantified for a 1 µl sample activated in 10 ml FSW using haemocytometer counts. A volume of diluted sperm solution (from the four pooled males of each treatment) required to achieve the appropriate sperm-to-egg ratio was added to each of the four egg suspensions to obtain four independent embryo populations with each possible parental cross: NFNM (naïve female gamete fertilized by naïve male gamete); NFEM (naïve female gamete fertilized by experienced male gamete); EFNM (experienced female gamete fertilized by naïve male gamete); EFEM (experienced female gamete fertilized by experienced male gamete). At 60 min post-fertilisation each concentrated embryo population was gently cleaned via serial partial water changes to remove any excess sperm, and diluted appropriately to obtain a density of approximately 80 eggs per ml in 20 l sterile plastic containers. Three replicate 1 ml samples from each population were fixed in 7% buffered formalin in seawater, and fertilisation success was scored within 3–4 hours as the proportion of eggs with a raised fertilisation envelope for 50–80 eggs per replicate. Temperature was maintained at the environmental ambient of approximately 15 °C by keeping the containers in a controlled-temperature room. Embryos were not fed as they were used within three days post-fertilisation and therefore remained at a pre-feeding stage throughout the experiment.

### PAH Exposure Experiment

Using a randomized block design, 36 plastic containers (2 l) were allocated a position in a controlled environment cabinet set on a 12 h day/night cycle and a constant temperature of 15 °C. Stock solutions of the PAH mixture were added to each experimental container to give final concentrations of 1080 ng/L for the high dose and 540 ng/L for the low dose with an acetone control. PAH mixtures and acetone for the controls were initially added to a small volume of FSW and left for 30 min for the acetone to evaporate off. Each container was then made up to 2 l with a volume of embryo solution calculated to give correct final PAH concentrations and to keep densities constant at 80 embryos per ml. At no stage were developing larvae fed so as to avoid the potentially confounding influence of their algal food. Three subsequent 1 ml subsamples were taken at 24, 48 and 72 h post-fertilisation, from each treatment group and fixed in 7% buffered formalin in seawater. Abnormality was scored within 3–4 hours by assessing the proportion of embryos (the grand mean of 40–60 per replicate) with normal development compared to those either dead, arrested at the one cell or cleavage stage, or that had undergone exogastrulation or had protruding cellular masses^59^. At the termination of the experiment (72 h post-fertilisation) the remaining embryo solutions in each jar were divided evenly into 4 subsamples (approximately 500 ml each), filtered through a mesh filter (80 µm) and the concentrated embryos stored in 1.5 ml Eppendorf tubes. These were snap frozen in liquid nitrogen and immediately stored in −80 °C for later biochemical analyses.

### Biochemical analyses

Adult gonad, released gametes and embryo tissues were analysed according to established biochemical methods^19, 25^. Details of these methods can be found in S1 *Materials and Methods*.

### Statistical analyses

Statistically significant differences (P < 0.05) in fertilisation success and gamete antioxidant markers among treatments were analysed using one-way ANOVA. Gonad oxidative stress markers and gamete damage markers were analysed by two-way ANOVA with parental PAH-exposure and sex as fixed factors. Embryo abnormality and oxidative stress markers were analysed across PAH treatments and among parental crosses by two-way ANOVA. Any statistically significant effects were further analysed using a posthoc Tukey HSD test. ANOVAs included tests for normality (Kolmogorov-Smirnov) and homogeneity of variance (Levene’s test). All analyses were performed using SigmaStat 2.03 (SPSS Inc.) or JMP (7.0) (SAS).

## Electronic supplementary material

Supplementary Information
