# Supplementary material for: Maternal antioxidant provisioning mitigates pollutant-induced oxidative damage in embryos of the temperate sea urchin Evechinus chloroticus
Source: Sci Rep. 2017 May 16;7:1954. doi: 10.1038/s41598-017-02077-5 (PMC5434045; doi:10.1038/s41598-017-02077-5)
Supplement: Supplementary file 1 — Supplementary Information [file 41598_2017_2077_MOESM1_ESM.pdf]

Manuscript title:

Maternal antioxidant provisioning mitigates pollutant-induced oxidative damage in embryos of the temperate sea urchin *Evechinus chloroticus*.

Authors:

Kathryn N. Lister[a*], Miles D. Lamare[b], David J. Burritt[a]

S1. Materials and Methods

PAH extractions

PAHs were extracted from *U. pertusa*, gonad and egg samples using the method of Burritt (1) with modifications. Briefly, PAHs were extracted from finely ground freeze-dried tissue with hexane:dichloromethane (85:15, v/v) using a ratio of 10 ml g^{-1} DW for 15 minutes with ultrasonication. Extracts were then centrifuged at 1500 g for 10 minutes and the supernatants passed through $NaSO_4$ columns (5 ml bed volume). Extracts were then evaporated to dryness for 24 hours in a fume cupboard, redissolved in 3 ml hexane and passed through silica columns (2 g of silica) using hexane:dichloromethane (1:1 v/v) as the elutant. The extracts were then evaporated to dryness and redissolved up to 1 ml of acetonitrile. Percentage recovery of selected C^{14} labelled PAHs from spiked samples was greater than 90% using this method.

PAH levels were determined by high performance liquid chromatography (HPLC) using a Perkin-Elmer liquid chromatography system, interfaced to a PC running Turbochrom software. The system consisted of a Series 200 pump, diode array, fluorescence detector and auto-sampler. PAHs were separated on an EnviroSep-PP 125 x 4.6 mm column (Phenomenix),

using water (A) and acetonitrile (B) as the mobile phases at a flow rate of 2 ml/min^{-1}. Separations were conducted according to the column manufacturers instructions for PAH separation (EPA 610) with starting conditions of A/B (60:40) run isocratically for 2 minutes after injection, followed by a linear gradient to 100% B over 25 minutes. PAHs were detected by UV at 245 nm and by programmed fluorescence; 0 minutes Exl: 240 nm Eml: 425 nm; 16 minutes Exl: 254 nm Eml: 395 nm. Authentic standards (Aldrich Chemical Co., Milwaukee, WI), dissolved in acetonitrile and run under the same conditions, were used for PAH identification and quantification. PAH detection limits ranged from 31 ng/g^{-1} DW for naphthalene to 1 ng/g^{-1} DW for chrysene.

Biochemical analyses

The activities of antioxidant enzymes and oxidative damage products were assessed in gonad, gamete and embryo tissue. Total protein was extracted for analysis of protein carbonyls and antioxidant enzyme activities by homogenising frozen gonad or muscle tissue samples (ground to a fine powder) in a 1:9 ratio (w/v) of 100 mM potassium phosphate buffer (pH 7.0) containing 0.1 mM Na_2 EDTA, 1% PVP-44, 1 mM PMSF and 0.5% v/v TritonX-100. Each homogenate was centrifuged at 13,000*g* for 15 minutes at 4°C, the supernatant decanted off and centrifuged a second time at 45,000*g* for 15 minutes at 4°C. Protein extracts were subjected to ultrafiltration using Porvair (Porvair Filtration Group Inc., Ashland, Virginia, USA) filtration plates (96-well 10KD MWCO) according to the manufacturer's instructions and reconstituted in 100 mM potassium phosphate buffer (pH 7.0). Supernatants were then collected and stored at -80°C until biochemical analysis. Soluble protein contents were determined using a Lowry protein assay as per Fryer et al. (2) with a bovine serum albumin (BSA) standard.

Protein carbonyl levels were determined in precipitated protein via reaction with 2.4-dinitrophenylhydrazine (DNPH) as described by Reznick and Packer (3). Superoxide dismutase (SOD; EC 1.15.1.1) was assayed using the microplate assay described by Banowetz et al. (4) with minor modifications. Catalase (CAT; EC 1.11.1.6) was assayed using the chemiluminescent method of Maral et al. (5), as adapted by Janssens et al. (6) for 96-well microplates. Glutathione reductase (GR; EC 1.8.1.7) was assayed using the method of Cribb et al. (7) with minor modifications. Glutathione peroxidase (GPx; EC 1.11.1.9) activity was measured according to the spectrophotometric method described by Paglia and Valentine (8). Glutathione-S-transferase (GST; EC 2.5.1.13) was determined using the method of Habig et al. (9), modified by Brogden and Barber (10) for use in a microplate reader. Activity of Glyoxalase-I (Glx-I; EC 4.4.1.5) was determined according to Hossain et al. (11) with minor modifications and activity of Glyoxalase-II (Glx-II; EC 3.1.2.6) was determined according to the method of Principato et al. (12), with minor modifications.

Glutathione was extracted by homogenising powdered tissue samples in a 1:10 ratio (w/v) of ice-cold 5% sulfosalicylic acid and centrifuged at 10,000 RPM for 15 minutes at 4°C. After centrifugation, supernatants were collected and stored at -80°C until biochemical analysis. Total glutathione plus glutathione disulfide (GSSG referred to as oxidised glutathione) levels were determined using the enzymatic recycling method employing the microplate assay described by Rahman et al. (13).

Lipids were extracted by homogenising powdered tissue samples in a 1:6 ratio (w/v) of methanol:chloroform (2:1 v/v) and leaving the suspension to stand for 1 minute at room temperature. Chloroform (400 µl) was added and the sample mixed by vortexing for 30 s. Deionised water (400 µl) was then added and the sample mixed by vortexing for 30 s. The phases were allowed to separate and 50 µl of the chloroform phase was collected and used for lipid hydroperoxide analysis. Lipid hydroperoxide levels were determined using the ferric thiocyanate method described by Mihaljevic et al. (14).

DNA was extracted, and checked for purity, from homogenised sample of sperm, egg and embryos using a BIOLINE Isolate II Genomic DNA kit according to the manufacturers instructions. Isolated DNA was collected and stored at -80°C until biochemical analysis. A sample of extracted DNA was precipitated by the addition of 0.1 volume of 4 M NaCl and 2.5 volumes of cold ethanol and digested as per Shigenaga et al (15) with modifications. Briefly, the precipitated DNA was re-dissolved in 200 ml of sterile DNA hydrolysis buffer (1 mM deferoxamine, 20 mM sodium acetate, pH 5). Nuclease P1 was added (4 µl; 3.3 mg ml^{-1}) and samples were incubated at 65°C for 15 minutes. Alkaline phosphatase (4 U in 1 M Tris-HCl (pH 8)) was added and the samples were incubated at 37°C for 60 minutes. Finally, 20 µl 3 M sodium acetate was added to each sample, followed by 20 µl of chelating solution (50 mM EDTA, 10 mM deferoxamine). The solutions were filtered through a 30 kDa cut-off filter-membrane and the filtered solutions, containing the nucleotides, were collected for 8-OHdG analysis. Digested DNA samples were analysed using high-performance liquid chromatography (HPLC) followed by UV detection of G and electrochemical detection (coulometric) of 8-OHdG. The procedure was performed essentially as described by Shigenaga et al. (15), using a C18 reverse-phase (5 mm, 4.6 mm x 250 mm) column (JASCO, Ishikawa-

cho, Hachioji-shi, Tokyo, Japan), a Perkin-Elmer HPLC system (Boston, U.S.A.) and an electrochemical detector (model 5100, ESA, Chelmsford, MA). The oxidation potentials of the analytical cell of the electrochemical detector were set to 150 mV and 350 mV for electrodes 1 and 2, respectively, with the guard cell potential set at 400 mV. Unmodified nucleosides were detected by their absorbance at 260 nm. Separation, of 50 ml of digested DNA, was achieved using an isocratic mobile phase consisting of 50 mM potassium phosphate (pH 5.5) and 10% methanol, at a flow rate of 1 ml min^{-1}, with the column maintained at 30°C. Peak data were collected and analyzed using a DataCenter 4000 general-purpose laboratory data interface, and Delta chromatography data acquisition and analysis software (DataworkX, Brisbane, Australia). The retention times for G and 8-OHdG were 12 and 17 minutes, respectively. Solutions of 8-OHdG and G (Sigma, Chemical Co, St Louis, MO, USA), prepared in HPLC-grade water (Merck, Darmstadt, Germany) and sterilised by passage through 0. 22 mm filters (Millipore, Bedford, MA, USA), were used as standards. For each sample, the amount of DNA injected onto the column was estimated using the signal for G, and 8-OHdG was quantified by comparison to external standards.

The microsomal fraction was obtained from gonad tissue as described by Ghosh et al. (16). Total protein was extracted and EROD activity was measured as described by Nahrgang et al. (17).

All assays were adapted for measurement using a microplate reader, with glass microplates used for the lipid hydroperoxide analysis, and were carried out using a PerkinElmer (Wallac) 1420 multilabel counter (Perkin Elmer, San Jose, California, U.S.A.) controlled by a PC, and fitted with a temperature control cell and an auto-dispenser. Data were acquired and processed using the WorkOut 2.0 software package (Perkin Elmer, San Jose, California, U.S.A.).

Table S1. ANOVA on concentrations of enzymatic defence markers superoxide dismutase (SOD), catalase (CAT), glutathione reductase (GR), glutathione peroxidase (GPx), glutathione-S-transferase (GST), glyoxalase-I (Glx-I), glyoxalase-II (Glx-II), total glutathione and percentage reduced glutathione in PAH-fed and control male and female *Evechinus chloroticus* gonad tissue. All analyses were two-way ANOVA with diet and sex as fixed factors.

Parameter	Source	df	SS	MS	*F*-value	*P*-value
SOD	Diet	1	28346.773	28346.773	37.44	**<0.001**
	Sex	1	9.030	9.030	0.01	0.915
	Diet x sex	1	5697.230	5697.230	7.53	**0.018**
	Residual	12	9084.971	757.081		
	Total	15	43138.004	2875.867		
CAT	Diet	1	55974.828	55974.828	46.22	**<0.001**
	Sex	1	22615.648	22615.648	18.67	**<0.001**
	Diet x sex	1	4394.364	4394.364	3.63	0.081
	Residual	12	14534.215	1211.185		
	Total	15	97519.055	6501.270		
GR	Diet	1	1.531	1.531	3.54	0.084
	Sex	1	1.458	1.458	3.37	0.091
	Diet x sex	1	0.052	0.052	0.12	0.735
	Residual	12	5.186	0.432		
	Total	15	8.227	0.548		
GPOX	Diet	1	1758.544	1758.544	54.24	**<0.001**
	Sex	1	642.116	642.116	19.81	**<0.001**
	Diet x sex	1	236.083	236.083	7.28	**0.019**
	Residual	12	389.069	32.422		
	Total	15	3025.812	201.721		
GST	Diet	1	581.172	581.172	5.19	**0.042**
	Sex	1	30.113	30.113	0.27	0.613
	Diet x sex	1	19.869	19.869	0.18	0.681
	Residual	12	1343.259	111.938		
	Total	15	1974.413	131.628		
Glx-I	Diet	1	20582.206	20582.206	4.83	**0.048**
	Sex	1	4446.889	4446.889	1.04	0.327
	Diet x sex	1	1713.546	1713.546	0.40	0.538
	Residual	12	51151.977	4262.665		
	Total	15	77894.618	5192.975		
Glx-II	Diet	1	131.618	131.618	7.78	**0.016**
	Sex	1	7.116	7.116	0.42	0.529
	Diet x sex	1	4.484	4.484	0.27	0.616
	Residual	12	202.927	16.911		
	Total	15	346.144	23.076		
Total glutathione	Diet	1	881.348	881.348	16.45	**0.002**
	Sex	1	4796.601	4796.601	89.54	**<0.001**
	Diet x sex	1	168.286	168.286	3.14	0.102
	Residual	12	642.825	53.569		
	Total	15	6489.059	432.604		
Reduced glutathione	Diet	1	324.991	324.991	12.58	**0.004**
	Sex	1	0.452	0.452	0.02	0.897
	Diet x sex	1	11.748	11.748	0.46	0.513
	Residual	12	310.113	25.843		
	Total	15	647.303	43.154		

Table S2. ANOVA on concentrations of enzymatic defence markers superoxide dismutase (SOD), catalase (CAT), glutathione reductase (GR), glutathione peroxidase (GPx), glutathione-S-transferase (GST), glyoxalase-I (Glx-I), glyoxalase-II (Glx-II), total glutathione and percentage reduced glutathione in eggs derived from PAH-fed and control female *Evechinus chloroticus*. All analyses were one-way ANOVA with parental diet as a fixed factor.

Parameter	Source	df	SS	MS	*F*-value	*P*-value
SOD	Parental diet	1	16081.833	16081.833	28.67	**0.006**
	Residual	4	2243.583	560.896		
	Total	5	18325.415			
CAT	Parental diet	1	39995.436	39995.436	527.30	**<0.001**
	Residual	4	303.397	75.849		
	Total	5	40298.833			
GR	Parental diet	1	5.881	5.881	83.35	**<0.001**
	Residual	4	0.282	0.071		
	Total	5	6.163			
GPOX	Parental diet	1	1219.800	1219.800	54.92	**0.002**
	Residual	4	88.850	22.213		
	Total	5	1308.650			
GST	Parental diet	1	216.000	216.000	8.84	**0.041**
	Residual	4	97.726	24.431		
	Total	5	313.726			
Glx-I	Parental diet	1	8936.672	8936.672	37.25	**0.004**
	Residual	4	959.545	239.886		
	Total	5	9896.217			
Glx-II	Parental diet	1	186.261	186.261	46.76	**0.002**
	Residual	4	15.934	3.983		
	Total	5	202.195			
Total glutathione	Parental diet	1	4452.650	4452.650	64.73	**0.001**
	Residual	4	275.138	68.785		
	Total	5	4727.788			
Reduced glutathione	Parental diet	1	21.433	21.433	0.34	0.591
	Residual	4	252.565	63.141		
	Total	5	273.998			

Table S3. ANOVA on concentrations of enzymatic defence markers superoxide dismutase (SOD), catalase (CAT), glutathione reductase (GR), glutathione peroxidase (GPx), glutathione-S-transferase (GST), glyoxalase-I (Glx-I), glyoxalase-II (Glx-II), total glutathione and percentage reduced glutathione in sperm samples derived from PAH-fed and control male *Evechinus chloroticus*. All analyses were one-way ANOVA with parental diet as a fixed factor.

Parameter	Source	df	SS	MS	*F*-value	*P*-value
SOD	Parental diet	1	1699.493	1699.493	1.62	0.272
	Residual	4	4199.356	1049.839		
	Total	5	5898.849			
GR	Parental diet	1	0.024	0.024	0.78	0.428
	Residual	4	0.124	0.031		
	Total	5	0.148			
GPOX	Parental diet	1	11.070	11.070	1.20	0.336
	Residual	4	37.039	9.260		
	Total	5	48.109			
Glx-I	Parental diet	1	0.150	0.150	0.01	0.931
	Residual	4	70.135	17.534		
	Total	5	70.285			
Total glutathione	Parental diet	1	12.127	12.127	0.52	0.51
	Residual	4	93.086	23.271		
	Total	5	105.212			

Table S4. ANOVA on oxidative damage markers (protein carbonyls, lipid hydroperoxides and 8-OHdG) in *Evechinus chloroticus* male and female gonad tissue, sperm and egg samples and 3-day old embryos derived from PAH-fed versus control parents. All analyses were two-way ANOVA with diet and sex as fixed factors for gonad and gamete analyses and treatment and parental cross as fixed factors for embryo analyses.

Parameter	Source	df	SS	MS	*F*-value	*P*-value
GONAD	Diet	1	7.223	7.223	29.30	**<0.001**
Protein	Sex	1	0.004	0.004	0.02	0.902
carbonyls	Diet x sex	1	0.000	0.000	0.00	0.988
	Residual	12	2.958	0.247		
	Total	15	10.185	0.679		
GONAD	Diet	1	38.720	38.720	89.45	**<0.001**
Lipid	Sex	1	8.688	8.688	20.07	**<0.001**
hydroperoxides	Diet x sex	1	1.238	1.238	2.86	0.117
	Residual	12	5.195	0.433		
	Total	15	53.840	3.589		
GAMETE	Diet	1	0.006	0.006	0.20	0.668
Protein	Gamete	1	0.004	0.004	0.12	0.738
carbonyls	Diet x gamete	1	0.003	0.003	0.10	0.762
	Residual	8	0.245	0.031		
	Total	11	0.258	0.023		
GAMETE	Diet	1	0.002	0.002	0.01	0.926
Lipid	Gamete	1	11.821	11.821	45.65	**<0.001**
hydroperoxides	Diet x gamete	1	0.013	0.013	0.05	0.83
	Residual	8	2.071	0.259		
	Total	11	13.907	1.264		
GAMETE	Diet	1	5.590	5.590	4.49	0.067
8-OHdG	Gamete	1	16.685	16.685	13.39	**0.006**
	Diet x gamete	1	0.085	0.085	0.07	0.801
	Residual	8	9.966	1.246		
	Total	11	32.326	2.939		
EMBRYO	PAH treatment	2	36.934	18.467	198.67	**<0.001**
Protein	Parental cross	3	11.780	3.927	42.24	**<0.001**
carbonyls	PAH treatment x parental cross	6	7.762	1.294	13.92	**<0.001**
	Residual	24	2.231	0.093		
	Total	35	58.706	1.677		
EMBRYO	PAH treatment	2	278.823	139.411	406.57	**<0.001**
Lipid	Parental cross	3	18.969	6.323	18.44	**<0.001**
hydroperoxides	PAH treatment x parental cross	6	22.281	3.713	10.83	**<0.001**
	Residual	24	8.229	0.343		
	Total	35	328.302	9.380		
EMBRYO	PAH treatment	2	64.107	32.053	46.78	**<0.001**
8-OHdG	Parental cross	3	6.283	2.094	3.06	**0.048**
	PAH treatment x parental cross	6	1.582	0.264	0.39	0.881
	Residual	24	16.445	0.685		
	Total	35	88.417	2.526		

Table S5. ANOVA on concentrations of enzymatic defence markers superoxide dismutase (SOD), catalase (CAT), glutathione reductase (GR), glutathione peroxidase (GPx), glutathione-S-transferase (GST), glyoxalase-I (Glx-I), glyoxalase-II (Glx-II), total glutathione and percentage reduced glutathione in PAH exposed embryos derived from PAH-fed and control *Evechinus chloroticus* parents. All analyses were two-way ANOVA with PAH treatment and parental cross as fixed factors.

Parameter	Source	df	SS	MS	*F*-value	*P*-value
SOD	PAH treatment	2	4967.667	2483.834	7.44	**0.003**
	Parental cross	3	75760.041	25253.347	75.67	**<0.001**
	PAH treatment x parental cross	6	3521.445	586.908	1.76	0.151
	Residual	24	8009.164	333.715		
	Total	35	92258.317	2635.952		
CAT	PAH treatment	2	7924.478	3962.239	6.48	**0.006**
	Parental cross	3	85688.628	28562.876	46.72	**<0.001**
	PAH treatment x parental cross	6	12783.884	2130.647	3.49	**0.013**
	Residual	24	14671.747	611.323		
	Total	35	121068.737	3459.107		
GR	PAH treatment	2	2.708	1.354	14.18	**<0.001**
	Parental cross	3	14.334	4.778	50.05	**<0.001**
	PAH treatment x parental cross	6	1.523	0.254	2.66	**0.04**
	Residual	24	2.291	0.096		
	Total	35	20.856	0.596		
GPOX	PAH treatment	2	568.242	284.121	12.10	**<0.001**
	Parental cross	3	3120.753	1040.251	44.30	**<0.001**
	PAH treatment x parental cross	6	324.326	54.054	2.30	0.067
	Residual	24	563.519	23.480		
	Total	35	4576.840	130.767		
GST	PAH treatment	2	191.072	95.536	2.85	0.078
	Parental cross	3	2014.478	671.493	20.00	**<0.001**
	PAH treatment x parental cross	6	20.435	3.406	0.10	0.996
	Residual	24	805.791	33.575		
	Total	35	3031.776	86.622		
Glx-I	PAH treatment	2	10629.865	5314.933	6.11	**0.007**
	Parental cross	3	108211.038	36070.346	41.45	**<0.001**
	PAH treatment x parental cross	6	2923.402	487.234	0.56	0.758
	Residual	24	20885.842	870.243		
	Total	35	142650.146	4075.718		
Glx-II	PAH treatment	2	84.626	42.313	7.15	**0.004**
	Parental cross	3	849.031	283.010	47.85	**<0.001**
	PAH treatment x parental cross	6	18.914	3.152	0.53	0.778
	Residual	24	141.962	5.915		
	Total	35	1094.533	31.272		
Total glutathione	PAH treatment	2	790.141	395.070	5.57	**0.01**
	Parental cross	3	14385.190	4795.063	67.60	**<0.001**
	PAH treatment x parental cross	6	361.008	60.168	0.85	0.546
	Residual	24	1702.482	70.937		
	Total	35	17238.821	492.538		
Reduced glutathione	PAH treatment	2	3473.067	1736.533	30.86	**<0.001**
	Parental cross	3	675.231	225.077	4.00	**0.019**
	PAH treatment x parental cross	6	503.809	83.968	1.49	0.223
	Residual	24	1350.674	56.278		
	Total	35	6002.782	171.508		

Table S6. ANOVA on abnormality following PAH exposure in three-day old *Evechinus chloroticus* embryos derived from PAH-fed versus control parents. Analyses were two-way ANOVA with PAH treatment and parental cross as fixed factors.

Parameter	Source	df	SS	MS	*F*-value	*P*-value
Abnormality	PAH treatment	2	67.646	33.823	0.79	0.464
24hrs	Parental cross	3	1434.424	478.141	11.22	**<0.001**
	PAH treatment x Parenatl cross	6	38.883	6.480	0.15	0.987
	Residual	24	1023.237	42.635		
	Total	36	2564.189	73.263		
Abnormality	PAH treatment	2	2491.592	1245.796	21.21	**<0.001**
48hrs	Parental cross	3	2546.024	848.675	14.45	**<0.001**
	PAH treatment x Parenatl cross	6	2224.659	370.777	6.31	**<0.001**
	Residual	24	1409.990	58.750		
	Total	36	8672.266	247.779		
Abnormality	PAH treatment	2	101.605	50.802	1.08	0.354
72hrs	Parental cross	3	1988.642	662.881	14.14	**<0.001**
	PAH treatment x Parenatl cross	6	1484.343	247.390	5.28	**0.001**
	Residual	24	1124.832	46.868		
	Total	36	4699.422	134.269		

References

1. Burritt DJ. The polycyclic aromatic hydrocarbon phenanthrene causes oxidative stress and alters polyamine metabolism in the aquatic liverwort *Riccia fluitans* L. Plant, Cell Environ. 2008;31(10):1416-31.
2. Fryer HJL, Davis GE, Manthorpe M, Varon S. Lowry protein assay using an automatic microtiter plate spectrophotometer. Anal Biochem. 1986;153(2):262-6.
3. Reznick AZ, Packer L. Oxidative damage to proteins - spectrophotometric method for carbonyl assay. Method Enzymol. 1994;233:357-63.
4. Banowetz GM, Dierksen KP, Azevedo MD, Stout R. Microplate quantification of plant leaf superoxide dismutases. Anal Biochem. 2004;332(2):314-20.
5. Maral J, Puget K, Michelson AM. Comparative study of superoxide-dismutase, catalse and glutathione peroxidase levels in erythrocytes of different animals. Biochem Biophys Res Commun. 1977;77(4):1525-35.
6. Janssens BJ, Childress JJ, Baguet F, Rees JF. Reduced enzymatic antioxidative defense in deep-sea fish. J Exp Biol. 2000;203(24):3717-25.
7. Cribb AE, Leeder JS, Spielberg SP. Use of a microplate reader in an assay of glutathione-reductase using 5,5'-dithiobis(2-nitrobenzoic acid). Anal Biochem. 1989;183(1):195-6.
8. Paglia DE, Valentine WN. Studies on quantitative and qualitative characterization of erythrocyte glutathione peroxidase. J Lab Clin Med. 1967;70(1):158-69.
9. Habig WH, Pabst MJ, Jakoby WB. Glutathione S-transferases - first enzymatic step in mercapturic acid formation. J Biol Chem. 1974;249(22):7130-9.
10. Brogdon WG, Barber AM. Microplate assay of glutathione S-transferase activity for resistance detection in single-mosquito triturates. Comp Biochem Physiol B: Biochem Mol Biol. 1990;96(2):339-42.
11. Hossain MA, Hasanuzzaman M, Fujita M. Up-regulation of antioxidant and glyoxalase systems by exogenous glycinebetaine and proline in mung bean confer tolerance to cadmium stress. Physiol Mol Biol Plants. 2010;16(3):259-72.

12. Principato GB, Rosi G, Talesa V, Giovannini E, Uotila L. Purification and characterization of 2 forms of glyoxalase-II from the liver and brain of Wistar rats. Biochimica Et Biophysica Acta. 1987;911(3):349-55.
13. Rahman I, Kode A, Biswas SK. Assay for quantitative determination of glutathione and glutathione disulfide levels using enzymatic recycling method. Nat Protoc. 2006;1(6):3159-65.
14. Mihaljevic B, KatusinRazem B, Razem D. The reevaluation of the ferric thiocyanate assay for lipid hydroperoxides with special considerations of the mechanistic aspects of the response. Free Radical Biol Med. 1996;21(1):53-63.
15. Shigenaga MK, Aboujaoude EN, Chen Q, Ames BN. Assays of oxidative DNA-damage biomarkers 8-oxo-2'-deoxyguanosine and 8-oxoguanine in nuclear-DNA and biological fluids by high-performance liquid-chromatography with electrochemical detection. Oxygen Radicals in Biological Systems, Pt D. 1994;234:16-33.
16. Ghosh R, Lokman PM, Lamare MD, Metcalf VJ, Burritt DJ, Davison W, et al. Changes in physiological responses of an Antarctic fish, the emerald rock cod (*Trematomus bernacchii*), following exposure to polybrominated diphenyl ethers (PBDEs). Aquat Toxicol. 2013;128:91-100.
17. Nahrgang J, Jönsson M, Camus L. EROD activity in liver and gills of polar cod (Boreogadus saida) exposed to waterborne and dietary crude oil. Mar Environ Res. 2010;70(1):120-3.
